# Involvement of Pharmacists in the Emergency Department to Correct Errors in the Medication History and the Impact on Adverse Drug Event Detection

**DOI:** 10.3390/jcm12010376

**Published:** 2023-01-03

**Authors:** Clara Goulas, Laura Lohan, Marion Laureau, Damien Perier, Véronique Pinzani, Marie Faucanie, Valérie Macioce, Grégory Marin, Isabelle Giraud, Maxime Villiet, Mustapha Sebbane, Cyril Breuker

**Affiliations:** 1 Clinical Pharmacy Department, CHU Montpellier, University of Montpellier, 34295 Montpellier, France; 2 PhyMedExp, University of Montpellier, CNRS, INSERM, 34000 Montpellier, France; 3 Emergency Medicine Department, CHU Montpellier, University of Montpellier, 34295 Montpellier, France; 4 Medical Pharmacology and Toxicology Department, CHU Montpellier, University of Montpellier, 34295 Montpellier, France; 5 Clinical Research and Epidemiology Unit, CHU Montpellier, University of Montpellier, 34295 Montpellier, France; 6 Economic Evaluation Unit, CHU Montpellier, University of Montpellier, 34295 Montpellier, France

**Keywords:** adverse drug event, medication history, emergency department, pharmacist

## Abstract

(1) Incomplete or wrong medication histories can lead to missed diagnoses of Adverse Drug Effects (ADEs). We aimed to evaluate pharmacist-identified ED errors in the medication histories obtained by physicians, and their consequences for ADE detection. (2) This prospective monocentric study was carried out in an ED of a university hospital. We included adult patients presenting with an ADE detected in the ED. The best possible medication histories collected by pharmacists were used to identify errors in the medication histories obtained by physicians. We described these errors, and identified those related to medications involved in ADEs. We also identified the ADEs that could not have been detected without the pharmacists’ interventions. (3) Of 735 patients presenting with an ADE, 93.1% had at least one error on the medication list obtained by physicians. Of the 1047 medications involved in ADEs, 51.3% were associated with an error in the medication history. In total, 23.1% of the medications involved in ADEs were missing in the physicians’ medication histories and were corrected by the pharmacists. (4) Medication histories obtained by ED physicians were often incomplete, and half the medications involved in ADEs were not identified, or were incorrectly characterized in the physicians’ medication histories.

## 1. Introduction

**Figure 1:**
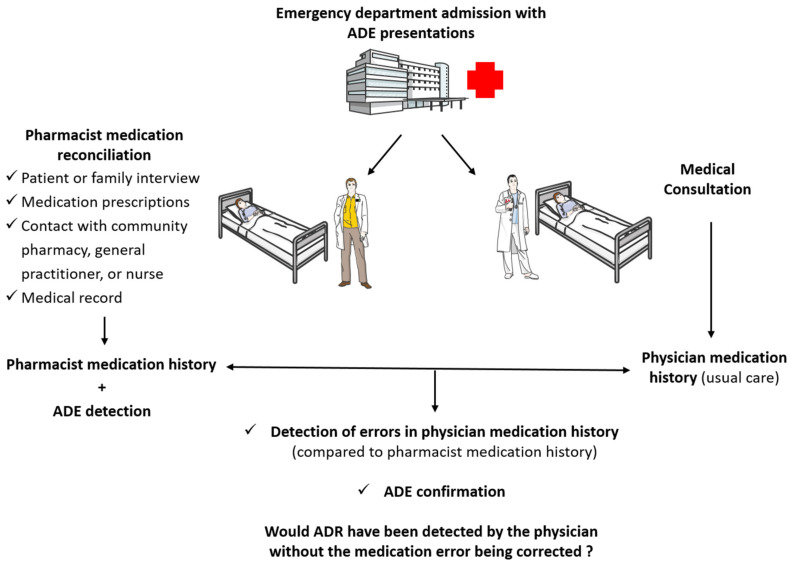
Principle of pharmacist medication history and detection of adverse drug events in the emergency department.

Adverse drug events (ADEs) are defined as any injury occurring to patients in relation to their medication management and resulting from appropriate care, inappropriate care or care deficit [1,2]. This definition includes injuries (signs, symptoms or laboratory abnormalities) resulting from adverse drug reactions (ADRs), or non-compliance with medication prescriptions [3]. Given their human and medico-economic impacts, and their role in unfavorable clinical evolution, ADEs are currently a major public health problem [4,5,6]. According to the literature, the ADE incidence ranges from 2 to 21% in inpatients [7], and from 5 to 35% in outpatients [8]. ADEs are the leading cause of unplanned admissions and death in hospitals [9,10,11]. According to the longitudinal, prospective study on ADE incidence (ENEIS-3) conducted in France in 2019, between 176,000 and 372,000 stays are caused annually by ADEs, including 93,000 to 197,000 stays caused by avoidable ADEs [12]. If we focus on emergency departments (ED), several teams that were trained to detect ADEs found up to 25% of patients with ADEs in EDs in France [13] and abroad [11]. In order to limit readmissions, it is necessary to detect these ADEs and review the drug treatments. However, ADEs are difficult to detect within the ED [14,15,16]. Most ED physicians have minimal to modest success in identifying ADEs [16,17]. One of the main reasons for this is incomplete or unavailable medication histories [18]. According to the literature, a vast majority of medication histories contain at least one error, particularly in EDs [19,20,21,22]. A loss of information about drug treatment between health professionals and the transition points in the patient’s care pathway, such as an outpatient’s entrance to the ED or the transfer of a patient from the ED to a hospital unit, is the main cause for these errors [14,23]. These errors in medication history prevent physicians from establishing the link between the symptomatology and the medication, and therefore, from detecting ADEs. Moreover, these medication errors pose a potential danger to the patient. According to the national drug safety agency (ANSM, *Agence Nationale de Sécurité du Medicament et des Produits de Santé*), a medication error is the unintentional omission or unintended performance of an act during the care process involving a drug, which may result in a risk or adverse event for the patient [24]. The introduction of medication reconciliation enables the correction of errors in medication histories, and thus both reduces the risk of medication errors and may improve ADE detection. It has been shown that pharmacists and pharmacist-technicians are among the most effective healthcare professionals for performing medication reconciliation [25,26,27,28,29]. It has also been shown that a clinical pharmacist positioned within the ED medical team enhances both the pharmaceutical and medical care to patients with varied complaints [30], and can improve ADE detection [13,31,32,33]; these professionals bring pharmaceutical expertise, and help ensure the production of a reliable medication history. In a cohort of patients who were admitted to the ED with ADE presentations, we compared medication histories obtained by physicians and pharmacists, and hypothesized that the medications missing from the physicians’ medication histories may well have resulted in missed diagnoses of ADE-related presentations (Figure 1).

Our primary objective was to assess errors in the medication lists obtained by physicians that may have led to a missed diagnosis of ADE if not corrected by pharmacists. Our secondary objective was to assess the transmission of information about ADE detection, and the involved medications at ED discharge. 

## 2. Materials and Methods

### 2.1. Study Design and Setting

**Figure 2:**
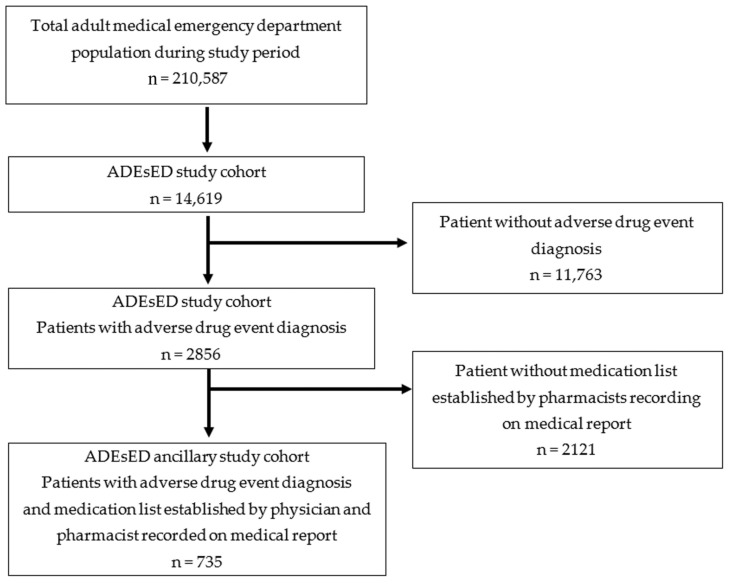
Flow chart of the study population. ADE, adverse drug event; ADEsED, adverse drug event detection at ED.

This study is ancillary to a study on ADE detection in EDs (ADEsED). This prospective observational monocentric study was conducted in the adult ED of a university hospital in France, from November 2011 to November 2018, and the primary objective was to assess the rate and characteristics of ADEs identified by the pharmacist team in an ED [34,35,36]. Participation in the study was proposed by clinical pharmacists for all adult patients (>18 years) admitted to the ED during the study period. Patients were prospectively and consecutively included, and were followed up until ED discharge. Patients were not included if they presented with acute psychological problems, or if they (or a family member) refused to participate in the study. The pharmaceutical team carried out structured interviews to determine the best possible medication history (BPMH), including self-medication and as required medications, and a medication reconciliation according to the High 5s project standard operating procedures (see description in the section, Errors on medication lists and ADE detection). Physicians also drew up medication lists after interviewing patients according to the usual care, without any standardized procedure. The medication lists collected from the clinical pharmacists and those collected from the medical team were obtained independently and blinded from each other. Since 2016, both types of lists are collected in the medical records, and it was during this same period that data about medication errors were collected. For this subgroup analysis, we extracted a cohort of patients with the following criteria: patients with ADE detected at ED admission, patients with an admission medication list established by both the pharmaceutical team and the medical team, and patients with medication error detection. Thus, we excluded from the ADEsED study cohort those patients without an ADE (*n* = 11,763), and those for whom a medication list established by the clinical pharmacy team was not recorded in the medical report (*n* = 2121) (Figure 2, flow chart).

### 2.2. Errors on Medication Lists and ADE Detection

Our process of medication reconciliation [37] and ADE detection has been previously published [34,35], and is briefly described below. The process is conducted by a clinical pharmacy team that includes one senior pharmacist, one pharmacist resident and four pharmacy students. All clinical pharmacy team members received specific training on taking medication histories according to the High 5s project standard operating procedures [38], and on ADE detection documentation [3,39]. The World Health Organization (WHO) initiated the Action on Patient Safety High 5s Project. The aim of this project was to achieve a significant reduction in five highly prevalent patient safety problems by applying standardized patient care processes. Medication reconciliation was chosen as one of these processes.

#### 2.2.1. Best Possible Medication History

The clinical pharmacy team carried out patient-structured interviews in the ED to determine the BPMHs (including self-medication and when-required medication) and self-reported adherence. If the interview could be conducted with the patient, it was conducted with a reliable person or family member. The BPMH is based on this interview and on additional sources, such as medication prescriptions, medical records, and contact with the community pharmacy, general practitioner or nurse. 

#### 2.2.2. Error Detection

The medication lists obtained by the pharmaceutical and medical teams were compared to identify errors. In agreement with the ED pharmacists and physicians, we defined errors on medication lists as any difference observed on the medication list obtained by the medical team as compared to the list obtained by pharmacists. These errors could be (i) an added medication; (ii) a missing medication due to medication omission or incorrect medication; or (iii) a medication mischaracterization, such as incorrect dosage, incorrect frequency, omission of dosage, omission of frequency, or omission of both frequency and dosage.

#### 2.2.3. Missed Diagnosis of ADE and Medication List Errors

To confirm our hypothesis that the medications identified by pharmacists enabled ADE detection, the relationship between medication list error and ADE detection was retrospectively evaluated by an expert committee that included ED physicians, clinical pharmacists and a pharmacovigilance physician. In order to limit the risk of bias, and in agreement with the expert committee, only errors concerning missing medication (omission and wrong molecule) involved in an ADE were analyzed. Indeed, the expert committee stated that mischaracterization errors (dosage or frequency) were not analyzed, and that the correction of the mischaracterization errors alone would not be sufficient to detect an ADE, but would contribute to the better detection of ADEs.

#### 2.2.4. ADE Detection

In our center, an ADE was defined as an injury resulting from a medication [1,2,31]. This definition included injuries (signs, symptoms or laboratory abnormalities) resulting from adverse drug reactions (ADRs) or non-compliance with medication prescriptions [3]. Voluntary medication poisoning was excluded from our ADE definition. All members of the clinical pharmacy team had received specific training in ADE detection, based on recommended methods such as the Naranjo tool [3,39,40]. They conducted an analysis of the patient’s medical file and the BPMH to detect a possible ADE based on chronological, semiological (symptoms, contributing factors, complementary examination results, etc.) and bibliographic objective criteria. The ADE severity was assessed according to the Common Terminology Criteria for Adverse Events as spontaneous regression, regression after symptomatic treatment, hospitalization with no life-threat, life-threatening risk, and death [40]. In order to detect ADEs, the name of the drug and its dose and intake frequency are necessary. ADEs were attributed to a medication by the pharmacist, and confirmed in real time by the treating senior ED physician. In the case of doubt about the diagnosis or the category of an ADE during the study, the case was reviewed by the expert committee. At the end of the study, ADE cases were verified by two expert clinical pharmacists and/or the expert committee, if necessary (Figure 1) [35].

#### 2.2.5. Transmission of Information

We examined the notifications of an ADE and the involved medication by reviewing the ED discharge reports. We considered that the ADE was notified if any evidence of an ADE suspicion, diagnosis or management was documented in the discharge report.

### 2.3. Data Collection

For each patient, we collected the following: demographic data (age and sex); the FRench Emergency Nurses Classification in Hospital (FRENCH); the main cause for the ED visit; medication lists obtained by the pharmaceutical and medical teams (name of medication, dosage, frequency of administration); number of daily medications; number and nature of the data sources used for the BPMH; medication involved in the ADE [name of medication and Anatomical Therapeutic Chemical (ATC) classification]; severity of the ADE (spontaneous regression, regression after symptomatic treatment, hospitalization with no life-threat, hospitalization with life-threatening risk, death and undetermined); symptoms of the ADE; and ED patient outcome (discharge, hospitalization, death). The FRENCH triage classification has five levels: 1: Immediately life-threatening; 2: Marked impairment of a vital organ, or imminently life-threatening, or functionally disabling traumatic lesion; 3: Functional impairment, or organic lesions likely to deteriorate within 24 h, or complex medical situation justifying the use of several hospital resources; 4: Stable, non-complex functional impairment or organic lesions, but justifying the urgent use of at least one hospital resource; and 5: No functional impairment or organic lesion justifying the use of hospital resources. For each error on the medication list, the medication class (according to the ATC classification system) and the type of medication error were analyzed.

### 2.4. Statistical Analysis

The data were described with percentages for categorical variables and means and standard deviations (SD) for quantitative variables. Variables of interest were compared with Student’s *t*-test.

### 2.5. Ethical Consideration

Our study was performed according to the World Medical Association Declaration of Helsinki, and was approved by the Montpellier University Hospital Institutional Review Board. Oral consent was obtained by a clinical pharmacist from all participants, or from a member of their family (written informed consent for participation was waived for this study by the Montpellier University Hospital Institutional Review Board). This study was registered on ClinicalTrials.gov (NCT03442010).

## 3. Results

### 3.1. Characteristics of the Study Population

During the study period, 210,587 ED visits were made, and 6.9% were managed by the pharmaceutical team. In total, 735 patients were included in our ancillary study. Baseline characteristics of our patients are presented in Table 1. The mean age was 74.3 ± 17.4 years, and the sex ratio was close to 1. The main causes for the ED visit were neurological disorders (20.3%), bleeding (13.7%) and falling (12.8%). The major ADE symptoms were bleeding (26.8%), metabolic disorders (15.5%) and neurological disorders (15.0%). In 42.2% of the cases, the ADE regressed after symptomatic treatment. The ED outcomes were discharge for 47.1% of our population, hospitalization for 51.6% and death for 1.4%. Our population (*n* = 735) was comparable to the total ADEsED population with an ADE (*n* = 2856) in terms of age, gender, FRENCH triage scale, number of daily medications, ADE severity and ED outcomes.

### 3.2. Medication Histories Obtained by the Pharmaceutical and Medical Teams

The BPMHs of the clinical pharmacy team were based on an average of 2.9 ± 0.6 different sources of information, described in Table 2. The daily medication lists contained significantly fewer medications when obtained by the medical team than by the clinical pharmacy team (6.6 ± 3.9 vs. 8.8 ± 4.1, *p* < 0.0001).

### 3.3. Medication Histories Obtained by the Pharmaceutical and Medical Teams

Nearly all patients (93.1%) had at least one error on the physicians’ medication lists as compared to those obtained by pharmacists. Moreover, in 57.1% of the population, at least one of the errors on the physicians’ medication lists concerned a medication involved in an ADE. The mean number of errors per patient was 5.7 ± 4.1 (Table 2). In total, 4186 errors were found. Characteristics of the errors on medication lists obtained by ED physicians are described in Table 3.

### 3.4. Errors on Medication Lists Obtained by the Medical Team and ADEs

Of the 1047 medications involved in an ADE, 51.3% were associated with an error on the medication lists obtained by ED physicians (Table 3). Those errors were mainly medication omissions (41.9%), frequency omissions (24.6%), and both dosage and frequency omissions (14.3%). In total, 23.1% of the medications involved in an ADE were not identified by the medical team (21.5% with the medication missing and 1.6% of wrong molecule) and were indicated by the pharmacist, allowing for the detection of the ADE. The analysis of these 242 errors (medication missing and wrong molecule) confirmed that, without their correction, the ADE would not have been detected. Table 4 summarizes the relationship between the missing medication on the physician medication list and the related ADE symptoms. The most frequent situations involved bleeding (*n* = 30) and disturbed consciousness/fall (*n* = 33) without knowledge of involved medications. For 28.2% of the medications involved in an ADE, the missing information (dose and/or frequency) was provided by the pharmacists, which contributed to a better detection of ADEs but was not sufficient alone to detect the ADE according to the expert committee. The medication ATC classifications most concerned by these errors were the neurological system (30.2%), blood and blood-forming organs (28.3%), and the cardiovascular system (17.5%). ADEs and the involved medications were mentioned in the ED discharge report in 41.0% (301/735) and 30.5% (224/735) of the cases, respectively.

## 4. Discussion

To the best of our knowledge, our study is the first to describe the thoroughness and accuracy of the medication histories of patients admitted to an ED along with the involvement of these medications in ADEs. We found that about half the medications involved in an ADE were associated with an error in the physician medication history. The medication reconciliation process conducted by the pharmacists was able to correct these errors and improve ADE detection. We found that 93.1% of the patients visiting the ED had at least one error in their physician medication history. A systematic literature review of medication history errors at admission to hospital reported an error rate of 27% to 83% when non-prescription drugs were included [21]. In EDs, 33–96% of ED medication lists contain at least one error [19,20,22,41,42,43,44,45]. These results are concordant with our findings; however, the studies that were carried out are not totally comparable to ours, since they differed in terms of methodology, definition of error and population. Indeed, some studies underestimated errors in the medication histories as a result of involving a young population with few daily medications [19,20,41,42,43,45], or only focusing on omissions without including frequency or dosage errors [20,44], or only accounting for prescribed medications [42]. Other studies used fewer sources of information to take the medication histories [22,41,43]. We found that most of the errors on the ED physicians’ medication lists compared to those on the ED pharmacists’ lists were omissions (48.1%), consistent with the literature data [20,21,23,41,42,43,45,46,47,48,49].

Our study has several original features: (i) we focused on a large cohort of patients with ADRs, using a robust prospective detection methodology and an exhaustive data collection that allowed for a complete description of the ADEs (symptoms, severity, etc.) and of the medication involved; (ii) we performed a complete analysis of the ME found and (iii) we evaluated the impact of these medication errors on the detection of ADEs. Thus, we presented innovative results which, to our knowledge, have not yet been described in the literature, on the impact of medication errors on the detection of ADEs, and the importance of their correction.

Indeed, 23.1% of the ADEs could not have been detected without a pharmacist’s intervention, because the ED physician had an incomplete medication list with an omission of the medication involved in the ADE, or an error in the molecule name. It appears obvious that ED medical teams have diagnostic and treatment priorities that prevent them from retrieving a complete medication history. Moreover, a pharmacist’s intervention may have contributed to better detection, with 28.2% more ADEs detected through the recovery of missing information on doses and frequencies of administration. In a previous study, our team highlighted the independent predictors for hospitalization following an ED visit. Thus, we identified some of the variables associated with treatment (polypharmacy, treatment by antineoplastic or immunomodulating agents, blood, systemic anti-infective and metabolism medications) and the presence of an ADE. These results point to the importance and relevance of collecting medication data in the context of an ED visit [35]. Furthermore, our population sample for this ancillary study (*n* = 735 patients with an ADE) was comparable to the total population with ADEs in the primary ADEsED study (*n* = 2856 patients with an ADE). Thus, extrapolating our results to the total population of the ADEsED study, we estimated that 658 and 798 ADEs would have been detected and better detected through the BPMHs of the clinical pharmacy team, respectively. The BPMHs of the ED pharmacists could be considered complete and reliable, given the mean number of different information sources (2.9 per BPMH), which were cross-referenced. To obtain an accurate medication history, cross-referencing information sources is crucial [37]. Moreover, considering the ED’s large influx of patients and the limited time to establish a medication history, the most relevant sources should be prioritized. Personal communication with the community pharmacist was identified as the most reliable, available and complete source of information [46,50]. This is consistent with our results showing that general practitioners were contacted in only 10.5% of the BPMHs, whereas community pharmacists were contacted in 77.0% of the histories. In this study, we highlighted a traceability problem concerning ADEs detected in the ED. Indeed, only 41.0% of the ADEs were mentioned in the ED discharge report, and the medication involved was mentioned in only 30.5%. The community–hospital link is essential to guarantee continuous and consistent care. The emergency physician intervenes in an acute situation, but is not the patient’s referring physician. Medical guidance and patient follow-up are responsibilities of the general practitioner, sometimes accompanied by the community pharmacist. Therefore, it is very important that the relevant information detected in the ED is transmitted to the patient’s referring healthcare professionals, who can review and adapt the treatment involved in the ADE. Other studies have shown that most discharge reports are incomplete [51,52] in terms of the ADEs. However, this information is essential to initiate a process of therapeutic revision that will prevent recidivism and readmission to the ED. Indeed, according to the ADE definitions (only adverse drug effect or any problem related to drug treatment) and methodologies used (detection during patient management or during retrospective analysis of the database), the rate of readmission to the ED after an initial admission related to an ADE is between 3.6% and 18.7% [53,54].

Our study has some limitations, mainly with its monocentric design. The lack of a control arm without pharmacist intervention does not allow us to make conclusions about the specific role of pharmacists to improve ADE detection. Thus, we only know that at least 23.1% of the ADEs would not have been detected by the physician without the intervention of the pharmacist. Our study also has strengths, primarily its prospective design, the large population, the specificity of our population (population with ADEs), and the number of observations of the outcome of interest. We should also note the rigorous approach we used in obtaining medication histories and detecting ADEs.

## 5. Conclusions

Our study demonstrates that medication histories retrieved by pharmacists in an ED are more complete than those made by ED physicians. Nearly all medication histories made by the ED physicians contained an error, compared to the pharmacists’ BPMHs. Reliable medication histories are an essential part of medication safety and ADE detection. A common computerized medical record with all drug prescriptions, educating patients to always have a copy of their prescription and an updated medical report with them, can save time and improve the quality of patient management and ADE detection. Our study shows that the intervention of a pharmacist can improve the detection of ADEs, and may well reduce the risk of readmission. We also point out a problem of information transmission to the community healthcare professionals concerning ADEs retrieved within the ED. Transition points in a patient’s care path are points of risk for medication errors, and the lack of communication between various health professionals increases the risk of these errors. To optimize and secure the care information between the ED and community healthcare professionals, clinical pharmacist should be an essential member of the ED team [55]. Since this study was conducted, we have changed our practices; we leave only the pharmacist’s medication history in the ED report in those cases where a pharmacist was present. Moreover, the pharmacist’s inclusion in the ED team for this study has been implemented permanently, and is now part of routine practice. 

## Figures and Tables

**Table 1:** Characteristics of the study population.

Characteristics	Total (*n* = 735)
Age (years)	74.3 ± 17.4
Gender, male	369 (50.2)
FRENCH triage scale	
Level 1	40 (5.4)
Level 2	111 (15.1)
Level 3	384 (52.2)
Level 4	136 (18.5)
Level 5	64 (8.7)
Main cause of ED visit	
Neurological disorders	149 (20.3)
Bleeding	101 (13.7)
Falling	94 (12.8)
Hepato-gastrointestinal disorders	91 (12.4)
Pulmonary disorders	71 (9.7)
Cardiovascular disorders	61 (8.3)
Alteration of the general condition	58 (7.9)
Abnormality of biological results	28 (3.8)
Metabolic disorders	20 (2.7)
Other	62 (8.4)
ADE symptoms	
Bleeding	197 (26.8)
Metabolic disorders	114 (15.5)
Neurological disorders	110 (15.0)
Cardiovascular disorders	84 (11.4)
Hepato-gastrointestinal disorders	75 (10.2)
Hematology and coagulation test abnormalities	75 (10.2)
Skin diseases	22 (3.0)
Fatigue/fall	21 (2.9)
Other	37 (5.0)
ADE severity	
Spontaneous regression	51 (6.9)
Regression after symptomatic treatment	310 (42.2)
Hospitalization with no life threat	288 (39.2)
Hospitalization with life-threatening risk	51 (6.9)
Death	1.5 (2.0)
Undetermined	20 (2.7)
ED visit outcome	
Discharge	346 (47.1)
Hospitalization	379 (51.6)
Death	10 (1.4)

Data are presented as mean ± SD, or *n* (%); ADEs, adverse drug events; ED, emergency department; FRench Emergency Nurses Classification in Hospital (FRENCH triage scale level; level 1: Immediately life-threatening; level 2: Marked impairment of a vital organ, or imminently life-threatening, or functionally disabling traumatic lesion; level 3: Functional impairment, or organic lesions likely to deteriorate within 24 h, or complex medical situation justifying the use of several hospital resources; level 4: Stable, non-complex functional impairment or organic lesions, but justifying the urgent use of at least one hospital resource; level 5: No functional impairment or organic lesion justifying the use of hospital resources).

**Table 2:** Best possible medication history, number of daily medications and medication reconciliation process.

	Total (*n* = 735)
Number of sources used by clinical pharmacy team	2.9 ± 0.6
Type of information sources used by clinical pharmacy team	
Personal source (patient, family or entourage)	557 (75.8)
Medication prescription (or general practitioner referral letter, nursing home emergency liaison record, ambulance sheet, medications brough in, etc.)	718 (97.7)
Computerized medical file	117 (15.9)
Community pharmacist	566 (77.0)
General practitioner	77 (10.5)
Nurse	97 (13.2)
Number of daily medications	
Medication list made by emergency physicians	6.6 ± 3.9
Medication list made by pharmacists	8.8 ± 4.1
Errors on medication lists	
Patients with at least one error	684 (93.1)
Patient with an error regarding at least one drug involved in an ADE	420 (57.1)
Number of errors per patient	5.7 ± 4.1

Data presented as mean ± SD, or *n* (%).

**Table 3:** Characteristics of errors on physician medication lists.

Total Medications *n* = 6108	Total Medications with Error *n* = 4186	Errors Concerning ADE Medication *n* = 537
Medication involved in errors		
Alimentary tract and metabolism	935 (22.3)	47 (8.8)
Blood and blood-forming organs	402 (9.6)	152 (28.3)
Cardiovascular system	838 (20.0)	94 (17.5)
Dermatological	64 (1.5)	0 (0)
Genito-urinary system and sex hormones	108 (2.6)	11 (2.0)
Systemic hormonal preparations	104 (2.5)	8 (1.5)
Anti-infective drugs for systemic use	155 (3.7)	38 (7.1)
Antineoplastic and immunomodulating agents	34 (0.8)	5 (0.9)
Muscular-skeletal system	124 (3.0)	10 (1.9)
Nervous system	1088 (26.0)	162 (30.2)
Respiratory system	206 (4.9)	8 (1.5)
Sensory organs	102 (2.4)	0 (0)
Other	26 (0.6)	2 (0.4)
Type of errors		
Added medication	350 (8.4)	NA
Missing medication *		
Omission	2014 (48.1)	225 (41.9)
Wrong molecule	71 (1.7)	17 (3.2)
Mischaracterization **		
Wrong dose	121 (2.9)	30 (5.6)
Wrong frequency	187 (4.5)	47 (8.8)
Dosage omission	103 (2.5)	9 (1.7)
Frequency omission	820 (19.6)	132 (24.6)
Both dosage and frequency omission	520 (12.4)	77 (14.3)

Data are *n* (%); ADE, adverse drug event; NA, not applicable (because the medication added to the physician medication list was not part of the medications taken by the patient, and therefore could not have led to an ADE); * errors whose correction by pharmacists allowed ADE detection; ** errors whose correction by pharmacists contributed to better ADE detection.

**Table 4:** Missing medications on the physician medication list, and related adverse drug event symptoms (*n* = 242).

Missing Medications on the Physician Medication List (Drug Classes)	ADE Symptoms Related to a Medication Missing on the Physician Medication List (*n*)
Alimentary tract and metabolism (*n* = 21)	Dysglycemia (*n* = 11)
	Hepato-gastrointestinal disorders (*n* = 6)
	Other (*n* = 4)
Blood and blood-forming organs (*n* = 40)	Bleeding (*n* = 30)
	Hematology and coagulation test abnormalities (*n* = 4)
	Other (*n* = 6)
Cardiovascular system (*n* = 40)	Dyskalemia (*n* = 10)
	Hypotension (*n* = 5)
	Dysnatremia (*n* = 4)
	Respiratory disorders (*n* = 4)
	Malaise (*n* = 4)
	Renal disorders (*n* = 4)
	Other (*n* = 9)
Anti-infective drugs for systemic use (*n* = 30)	Hepato-gastrointestinal disorders (*n* = 8)
	Hematology and coagulation test abnormalities (*n* = 6)
	Allergy (*n* = 5)
	Other (*n* = 11)
Nervous system (*n* = 81)	Disturbed consciousness/fall (*n* = 33)
	Hepato-gastrointestinal disorders (*n* = 16)
	Dysnatremia (*n* = 8)
	Seizure (*n* = 4)
	Other (*n* = 20)
Genito-urinary system and sex hormones/Systemic hormonal preparations/Antineoplastic and immunomodulating agents/Muscular-skeletal system/Respiratory system/Other (*n* = 30)	Dyskalemia (*n* = 4)
	Dysglycemia (*n* = 3)
	Hypotension (*n* = 3)
	Other (*n* = 20)

Data are *n*; ADEs, adverse drug events.

## Data Availability

The data analyzed during the current study are not publicly available due ethical restrictions, but are available from the corresponding author on reasonable request.
